# Serum CCAT2 as a biomarker for adjuvant diagnosis and prognostic prediction of cervical cancer

**DOI:** 10.1186/s13048-022-00950-0

**Published:** 2022-02-03

**Authors:** Xiaoli Cao, Juan Yao, Meiqun Jia, Xianjuan Shen, Jinye Zhang, Shaoqing Ju

**Affiliations:** 1 Department of Laboratory Medicine, Affiliated Tumor Hospital of Nantong University, #48 West Qingnian Road, Nantong, 226019 Jiangsu Province China; 2 Gynecology of Affiliated Tumor Hospital of Nantong University, #48 West Qingnian Road, Nantong, 226019 Jiangsu Province China; 3 Laboratory Medicine Center, Affiliated Hospital of Nantong University, #20 Xisi Road, Nantong, 226001 Jiangsu Province China

**Keywords:** Cervical carcinoma, lncRNA CCAT2, Real-time quantitative PCR

## Abstract

Growing evidence indicates that lncRNA colon cancer-associated transcript 2 (CCAT2) is associated with cancers. However, the clinical value of CCAT2 in cervical cancer (CC) remains unclear. In this study, serum CCAT2 level was detected by real-time quantitative PCR (RT-qPCR). Carbohydrate antigen 125 (CA125) and squamous-cell carcinoma antigen (SCC) were detected by electrochemiluminescence. A receiver operating characteristic (ROC) curve was utilized to estimate the diagnostic efficiency of CCAT2. Kaplan-Meier survival analysis and univariable and multivariable analyses were performed to assess the prognostic value of CCAT2. The relative expression level of CCAT2 in primary CC patients was significantly higher than that in cervical intraepithelial neoplasias (CIN) patients and healthy controls (both *P <* 0.001). CCAT2 relative expression was positively correlated with tumor Federation of Gynecology and Obstetrics (FIGO) stage, SCC-Ag and lymph node metastasis (LNM) (all *P <* 0.05). CCAT2 expression in recurrent/metastatic CC was significantly higher compared with primary CC (*P* < 0.0001) or operated CC (*P* < 0.0001) and during follow-up, CCAT2 expression was increased before surgery and decreased significantly after surgery (*P* < 0.0001). Furthermore, the overall survival rate of CC patients with high CCAT2 expression group markedly decreased as compared with that of low CCAT2 expression group (*P* = 0.026). Univariate analyses indicated that CCAT2 was a poor prognostic factor associated with overall survival (OS). Our study indicates that CCAT2 may be valuable in complementary diagnosis and monitoring of progression and prognosis of CC patients. Combined detection of CCAT2, CA125 and SCC can greatly improve the diagnostic efficiency of primary CC.

## Introduction

Cervical cancer (CC) ranks the second in female malignant tumors, and the second cause of cancer-related death in cancer patients aged 20–39 years. In addition, the incidence of CC tends to increase gradually each year [1]. Early surgical intervention, radiotherapy and chemotherapy can offer a high survival rate in CC patients. However, many CC patients have already been in the middle or late state and lost the chance of surgery at the time of diagnosis. Radiochemotherapy for such patients is often ineffective and their survival and prognosis are usually poor. With technical advances in recent years, tumor markers have gradually been used for early screening of malignant tumors due to simplicity, microinvasiveness and quickness, and play a significant role in early diagnosis, therapeutic assessment and prognostic prediction of cancer patients.

Carcinoembryonic antigen (CEA), carbohydrate antigen 125 (CA125) and squamous cell carcinoma antigen (SCC) are three main tumor markers for early screening, therapeutic monitoring and prognostic assessment of CC at present. But as they are not sensitive and specific as expected, more reliable, sensitive and specific serum markers are required.

LncRNA colon cancer associated transcript 2 (CCAT2) is highly expressed in colon cancer and can promote tumor growth, metastasis and chromosomal instability. He et al. [2] found CCAT2 promoted prostate cancer cell proliferation and invasion by regulating the Wnt/β-catenin signaling pathway. Sun et al. [3] reported that low expression of miR-424 in normal human astrocytes (NHA) was accompanied with a high expression of CCAT2 and vascular endothelial growth factor-A (VEGFA). Xu et al [4] discovered that CCAT2 promoted the development and progression of triple-negative breast cancer (TNBC) by up-regulating the expression of OCT4-PG1 and activating Notch signaling. Roxana et al. [5] first analyzed the expression of CCAT2 in normal breast tissue and breast cancer tissue by RT-qPCR and observed that breast cancer patients with high CCAT2 expression could not benefit from cyclophosphamide (CTX) + 5-fluorouracil (5-FU) + methotrexate (CMF) adjuvant chemotherapy, and these patients often had a shorter survival duration. CCAT2 expression in liver cancer tissue was significantly higher than that in normal control. High CCAT2 expression is an independent risk factor of predicting shorter survival of hepatocellular carcinoma (HCC) patients [6].

We hypothesize that there is a correlation between CCAT2 and CC. In the present study, we explored CCAT2 expression in CC and evaluated the value as a serum biomarker for clinical adjuvant diagnosis and prognostic prediction of CC.

## Materials and methods

### Sample collection

Serum samples were collected from 180 primary CC patients aged 28–76 years with a median of 52 (45.0, 58.0) years, 165 postoperative CC patients aged 28–76 years with a median of 51 (46.0, 58.0) years, 44 inoperable recurrent and metastatic CC patients aged 31–70 years with a median of 54 (49.0, 60.0) years, 80 CIN patients aged 23–74 years with a median of 47 (40.0, 51.5) years, and 100 healthy individuals aged 24–69 years with a median of 48 (40.0, 56.0) years as control. In addition, serum samples were collected consecutively from 30 of the 180 primary CC patients who underwent operation during the follow-up periods of 1–180 days. The primary CC patients and CIN patients were selected from the patients who were clinic pathologically confirmed as having CC and received treatment in the department of gynecology of Nantong Tumor Hospital between January 2015 and December 2018. The healthy controls were selected from individuals who underwent physical examination in the PE Center of the same hospital during the same period. Tumor staging and typing were according to the American Joint Committee on Cancer (AJCC) guidelines [7]. This research protocol was approved by the ethics committee of the said hospital (LW2020002), and all samples were anonymous. Informed consent was obtained from all patients and controls. The blood samples were from the remaining samples that used for routine examination. Serum samples (5 ml each) were centrifuged at 1000 r/min for 10 min to collect the sera, which were restored at 80 °C for use.

### RNA extraction and cDNA synthesis

Serum total RNA was extracted using the serum RNA extraction kit (Beijing Patek Biotechnology Co., Ltd., Beijing, China). RNA optical density (OD) was measured by ultraviolet spectrophotometry to calculate the OD_260_/OD_280_ ratio of the RNA samples, knowing that the ratio between 1.8 and 2.0 indicates good purity of the RNA extracted. The extracted RNA was reverse transcribed into cDNA by using Revert Aid First Strand cDNA Synthesis Kit (Thermo, USA). The reaction system is as follows: 300 ng RNA, 4 μl 5× reaction buffer, 2 μl (10 nM) deoxynucleoside triphosphate (dNTP), 1 μl oligonucleotide as the primer (dT), 1 μl (200 U /μl) reverse transcriptase ribonuclease inhibitor, and addition of enzyme-free H_2_O to 20 μl. They were mixed thoroughly, centrifuged and reverse transcribed at 42 °C for 60 min and 70 °C for 5 min.

### RT-qPCR assay

RT-qPCR assay was performed with Light Cycler 480 RT-QPCR using the following reaction system: 10 μl SYBR Green I mix, 3 μl cDNA,1 μl up-stream primer, 1 μl down-stream primer, and 5 μl enzyme-free H_2_O to a total volume of 20 μl under the reaction condition of 95 °C for 5 min, one cycle; 95 °C, 15 s; 61 °C, 30s; 72 °C, 30s, totaling 45 cycles. Three wells were set for each sample and the mean value was used for analysis. lncRNA relative expression (RQ) = 2^-△△CT^ was used to indicate the relative expression of CCAT2, and △△ cycle threshold (Ct) = study group (CT_CCAT2_-CT_GAPDH_)--control group (CT_CCAT2_-CT_GAPDH_) was used as the mean value. The primer sequences are as follows: CCAT2 up-stream primer: 5′-CCCTGGTCAAATTGCTAAACCT-3′, down-stream primer: 5′-TTATTCGTCCCTCTGTTTTATGGAT-3′; GAPDH up-stream primer: 5′-TGATGACATCAAGAAGGTGGTGAAG-3′, down-stream primer: 5′-TCCTTGGAGGCCCAGTGGGCCAT-3′.

### Methodological evaluation

CA125 and SCC were detected by electrochemiluminescence using the E601 electrochemiluminescence instrument (Roche, Germany) and Maglumi automatic chemiluminescence instrument (New industry Biomedical Engineering Co., Ltd., Shenzhen, China) respectively.

### Follow-up

We collected information on 5-year survivors from 154 of the 180 primary CC patients. Follow-up with all CC patients occurred by telephone once every 3 months in the first 2 years and every 6 months after that.

### Statistical analysis

Statistical analysis and ROC curve mapping were performed by SPSS20.0. Pair-wise comparison of CCAT2 relative expression between primary CC patients, CIN patients and healthy controls was performed by Mann-Whitney U test. The correlation between CCAT2 relative expression and clinicopathological features was analyzed by χ^2^ test. OS was analyzed by Kaplan-Meier analysis. Univariate and multivariate analyses were evaluated with Cox proportional hazards models. Mapping was performed by GraphPad Prism 5. Statistically significant difference was set as *P* < 0.05.

## Results

### Serum relative expression of CCAT2 in primary patients, CIN patients and healthy controls

**Fig. 1:**
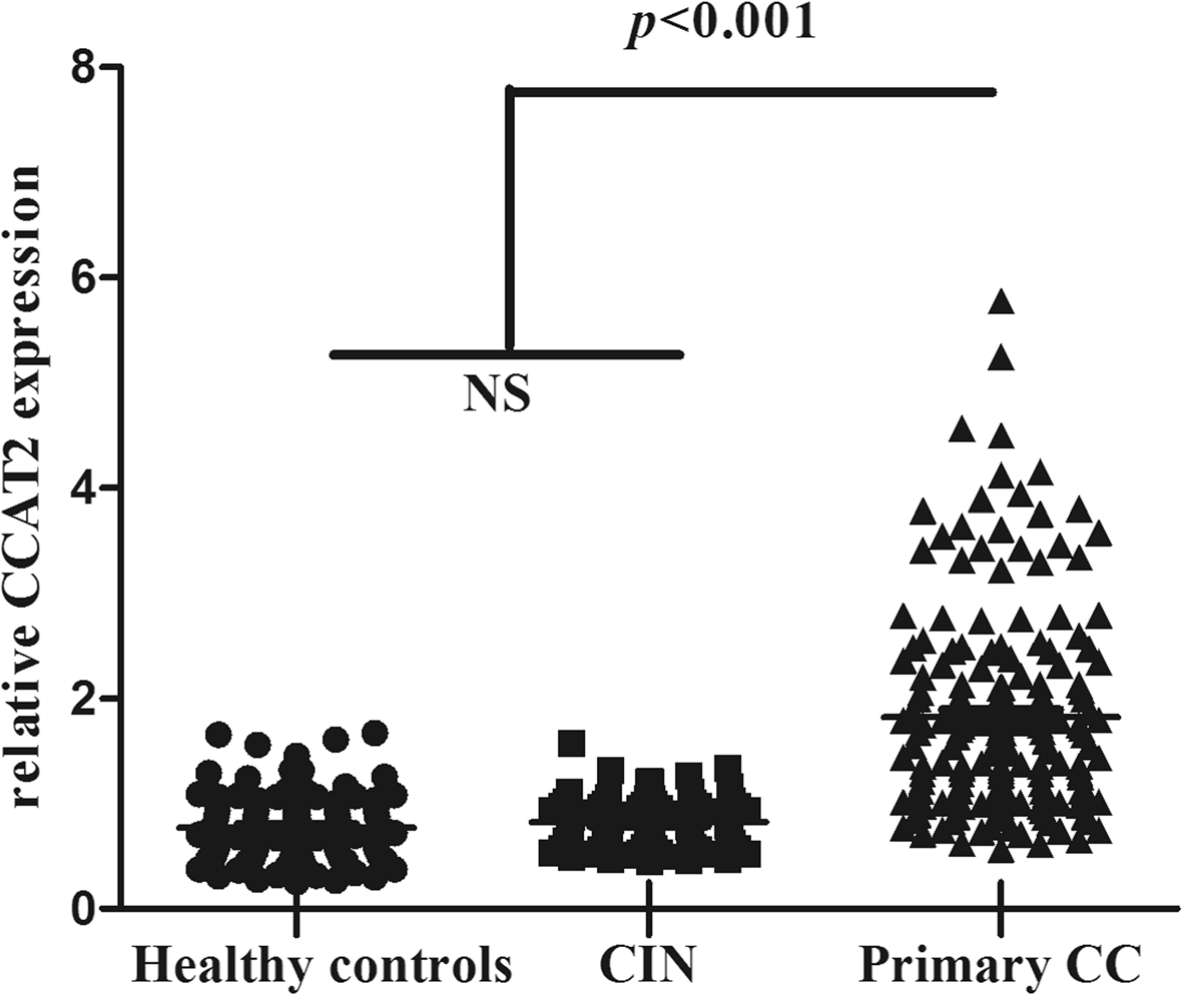
A scatter diagram of serum CCAT2 in healthy controls, CIN patients and primary CC patients. CCAT2 was detected by RT-qPCR. Pair-wise comparison of the relative expression of CCAT2 between primary CC patients, CIN patients and healthy controls was performed by Mann-Whitney U test, and *P* < 0.05 was considered statistically significant. NS: no significance

The median value of serum CCAT2 relative expression in the 100 healthy controls was 0.727 (0.544, 0.990) and 0.795 (0.591, 0.979) in the 80 CIN patients. There was no significant difference in serum CCAT2 relative expression between CIN patients and healthy controls (*P >* 0.05). The median value of serum CCAT2 relative expression in 180 primary CC patients was 1.616 (1.068, 2.219), which was significantly higher than that in CIN patients and healthy controls (both *P <* 0.001) (Fig. 1).

### Correlation between serum CCAT2 relative expression and clinicopathological features of primary CC patients

Correlation analysis of serum CCAT2 relative expression with age, menopause, tumor size, FIGO stage, pathological type, SCC-Ag and the presence or absence of LNM in 180 primary CC patients showed that serum CCAT2 relative expression was positively correlated with tumor FIGO stage, SCC-Ag and LNM (all *P <* 0.05) (Table 1).

**Table 1:** Correlation between serum CCAT2 relative expression and clinicopathological features of CC patients

Clinicopathologicfeatures	*n*=	CCAT2 relative expression	*P value*
		[median (upper and lower quartile)]	
Total (*n*)	180		
Age (yr)			0.222
≤50	79	1.434 (1.050, 2.343)	
>50	101	1.679 (1.079, 2.131)	
Menopause			0.460
yes	94	1.586 (1.043, 2.282)	
no	86	1.675 (1.110, 2.219)	
Tumor size			0.101
≤4cm	90	1.357 (1.021, 2.129)	
>4cm	90	1.749 (1.181, 2.343)	
FIGO stage			0.038*
IA1-Ib1	63	1.283 (1.007, 2.056)	
Ib2-IIa2	112	1.722 (1.189, 2.298)	
≥IIb	5	3.411 (1.052, 3.822)	
Pathological type			0.263
aquamous Ca	152	1.675 (1.110, 2.131)	
adenal Ca	22	1.503 (1.021, 3.541)	
adenosqua. Ca	6	1.153 (0.924, 1.357)	
SCC-Ag (ng/ml)			0.001*
negative	98	1.352 (1.014, 1.866)	
positive	82	1.886 (1.266, 2.453)	
LNM			0.046*
negative	150	1.517 (1.047, 2.129)	
positive	30	1.904 (1.257, 2.596)	

*P<0.05; FIGO: International Federation of Gynecology and Obstetrics; *CC* Cervical cancer *CCAT2* Colon cancer-associated transcript 2, *LNM* Lymph node metastasis

### Application of CCAT2, CA125 and SCC detection to diagnosis of primary CC

Pairwise comparison demonstrated that the serum concentration of CA125 in 180 primary CC patients was 16.14 (10.55, 23.08) U/ml vs. 11.50 (7.53, 16.50) U/ml in 100 healthy controls, showing a significant difference between the two groups (*P <* 0.05). The serum concentration of SCC and CCAT2 in primary CC patients was 2.10 (1.20, 6.60) ng/ml vs. 0.90 (0.60, 1.30) ng/ml in healthy controls and 1.616 (1.068, 2.219) vs. 0.727 (0.544, 0.990) respectively, showing significant differences between the two groups (*P <* 0.001) (Table 2).

**Table 2:** The median and cutoff values of CCAT2, CA125 and SCC in primary CC patients and healthy controls

Parameters	Primary CC	Healthy controls	Cutoff	AUC	*p*
CCAT2	1.616 (1.068, 2.219)	0.727 (0.544, 0.990)	1.102	0.897	<0.001
CA125 (U/ml)	16.14 (10.55,23.08)	11.50 (7.53,16.50)	9.69	0.678	*<*0.05
SCC (ng/ml)	2.10 (1.20,6.60)	0.90 (0.60,1.30)	1.55	0.815	<0.001

**Fig. 2:**
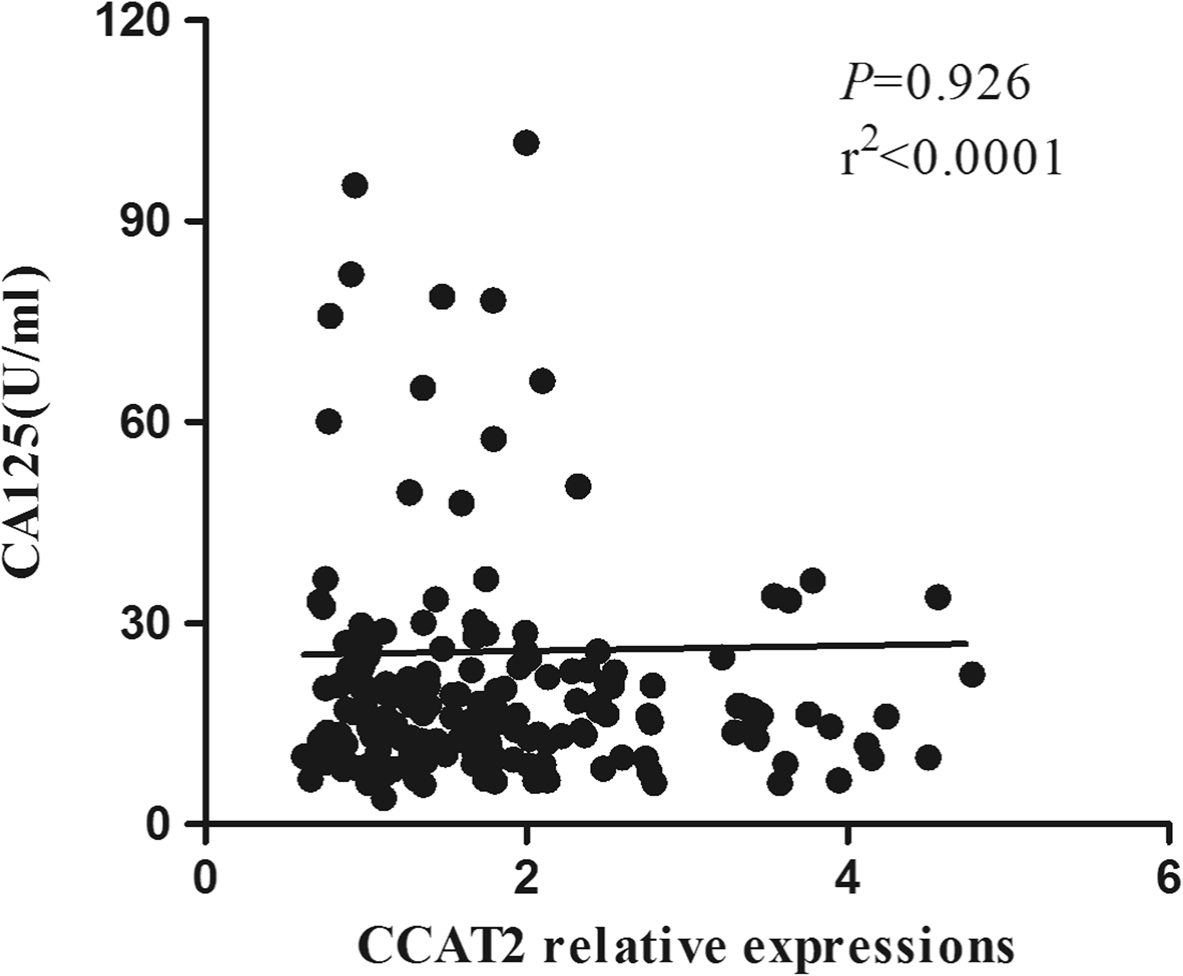
A scatter diagram of CCAT2 and CA125 correlation in primary CC patients, *P* = 0.926, *r*^2^ < 0.0001

**Fig. 3:**
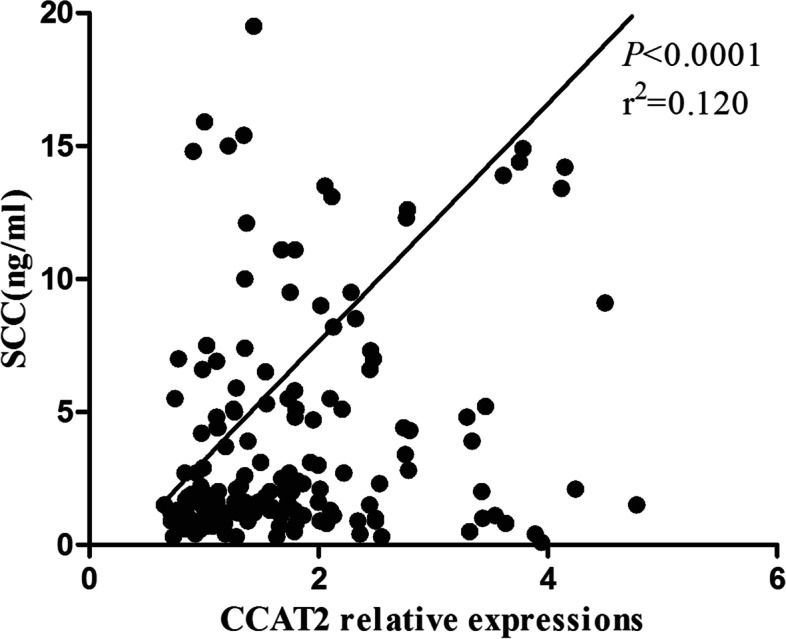
A scatter diagram of CCAT2 and SCC correlation in primary CC patients, *P* < 0.0001, *r*^2^ = 0.120

Serum CCAT2 relative expression was not significantly correlated with the CA125 concentration in 180 primary CC patients (*P* = 0.926, *r*^2^ < 0.0001) but significantly correlated with the SCC concentration (*P* < 0.0001, *r*^2^ = 0.120) (Figs. 2, 3).

### Evaluation of the diagnostic value of serum CCAT2, CA125 or SCC for primary CC

**Fig. 4:**
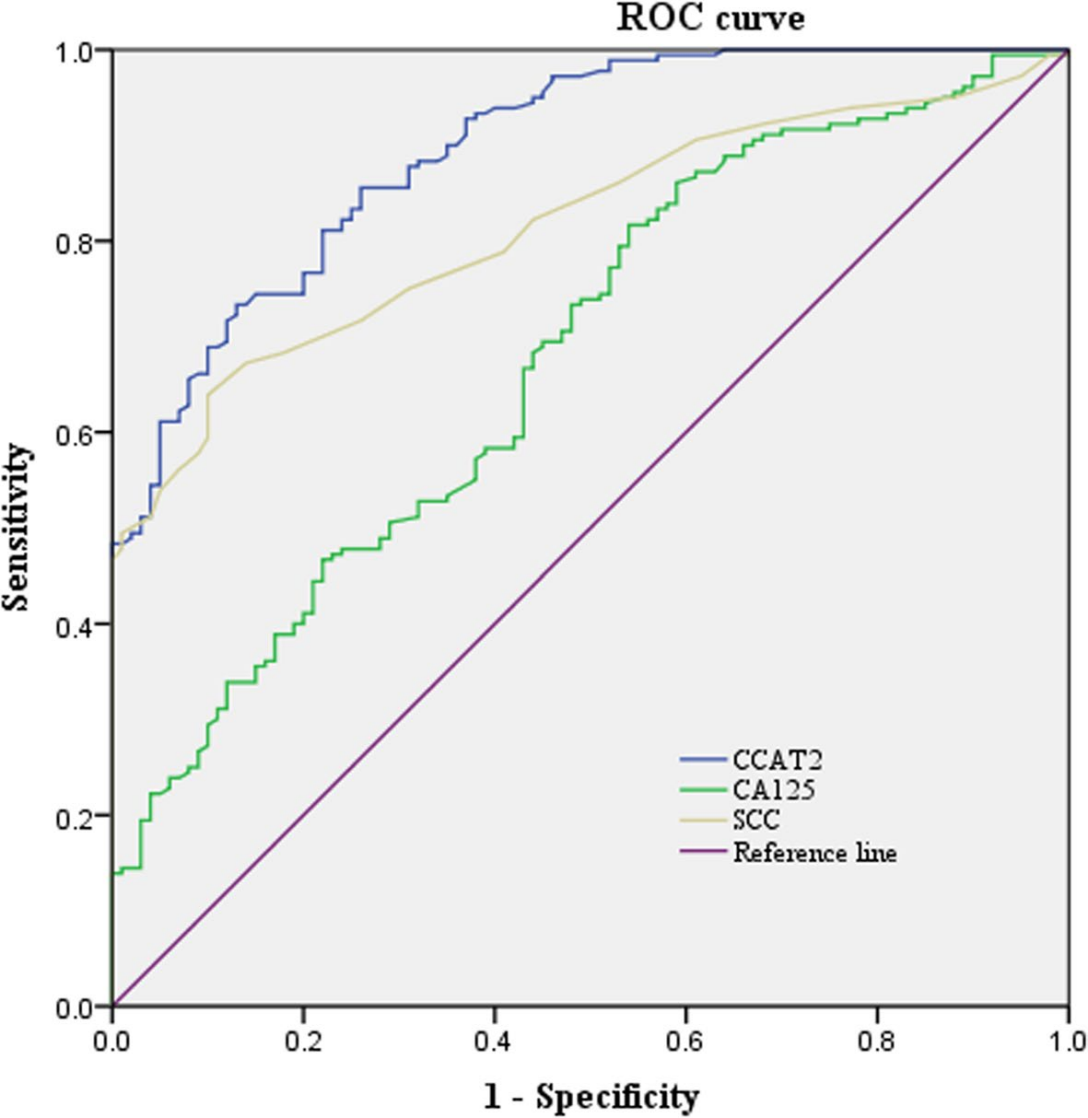
RCO differentiation between primary CC patients and healthy controls. The AUC value of CCAT2, CA125 and SCC was 0.897 (95%CI: 0.862–0.933), 0.678 (95%CI:0.614–0.743) and 0.815 (95%CI:0.767–0.863), respectively

When the cutoff value of CCAT2 for diagnosing CC was 1.102 (73.33%sensitivity and 87.00% specificity), the AUC (area under the curve) for CCAT2 was 0.897 (95%CI: 0.862–0.933). By that analogy, the cutoff value used for CA125 was 9.69 U/ml (81.67% sensitivity and 46.00% specificity). The AUC for CA125 was 0.678 (95%CI: 0.614–0.743). The cutoff value used for SCC was 1.55 ng/ml (69.44% sensitivity and 90.00% specificity). The area under the curve for SCC was 0.815 (95%CI: 0.767–0.863) (Fig. 4).

Next, a combined analysis of CCAT2, CA125 and SCC was performed using a tandem model of the three markers. The sensitivity, specificity, accuracy, positive prediction and negative prediction of this panel in differentiating the healthy controls were 94.44, 96.00, 95.00, 97.70 and 90.57% respectively, which greatly improved the diagnostic efficiency in diagnosing CC (Table 3).

**Table 3:** The value of CCAT2, CA125 and SCC in diagnosing primary CC

Molecular marker	Sensitivity	Specificity	Accuracy	Positive prediction	Negative prediction
	(%)	(%)	(%)	(%)	(%)
CCAT2	73.33	87.00	78.21	91.03	64.44
	(132/180)	(87/100)	(219/280)	(132/145)	(87/135)
CA125	81.67	46.00	68.93	73.13	58.23
	(147/180)	(46/100)	(193/280)	(147/201)	(46/79)
SCC	69.44	90.00	76.79	92.59	62.07
	(125/180)	(90/100)	(215/280)	(125/135)	(90/145)
CCAT2+CA125	91.67	89.00	90.71	93.75	85.58
	(165/180)	(89/100)	(254/280)	(165/176)	(89/104)
CCAT2+SCC	82.22	96.00	87.14	97.37	75.00
	(148/180)	(96/100)	(244/280)	(148/152)	(96/128)
Three combination	94.44	96.00	95.00	97.70	90.57
	(170/180)	(96/100)	(266/280)	(170/174)	(96/106)

### CCAT2 relative expression in postoperative CC patients and recurrent/metastatic CC patients

**Fig. 5:**
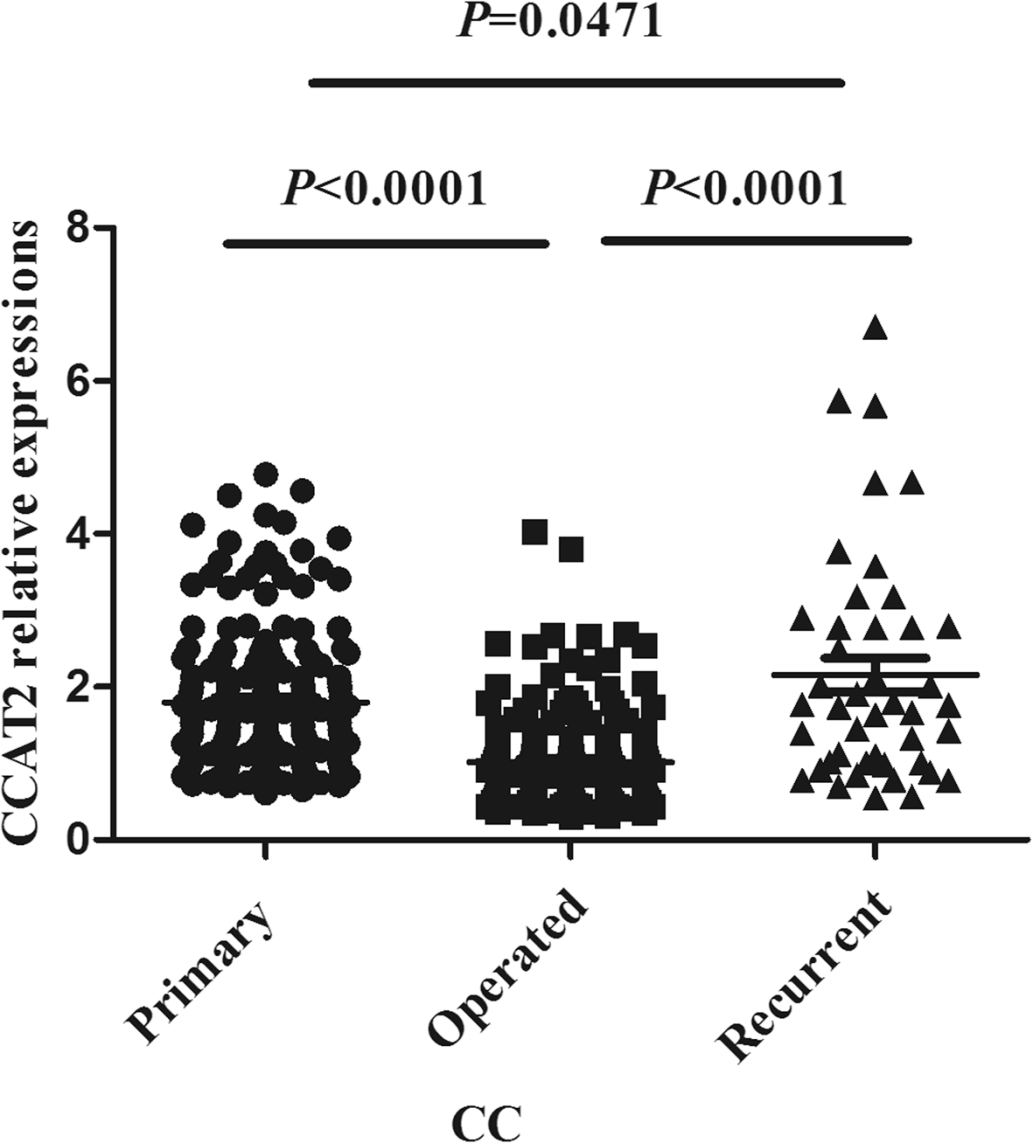
A scatter diagram of CCAT2 relative expression in primary CC patients, postoperative CC patients, and recurrent/metastatic CC patients. Man-Whiney U test, *P* < 0.05; the horizontal line indicates the median line of each group

The median value of serum CCAT2 relative expression in 165 postoperative CC patients was 0.790 (0.607, 1.165), showing a significant difference from that in primary CC patients (*P* < 0.0001) (Fig. 5). The median value of serum CCAT2 relative expression in 44 recurrent and metastatic CC patients was 1.752 (1.003, 2.787), showing a significant difference from that in primary CC patients and postoperative CC patients (*P* = 0.0471, *P* < 0.0001) (Fig. 5).

### CCAT2 dynamic in postoperative CC patients

**Fig. 6:**
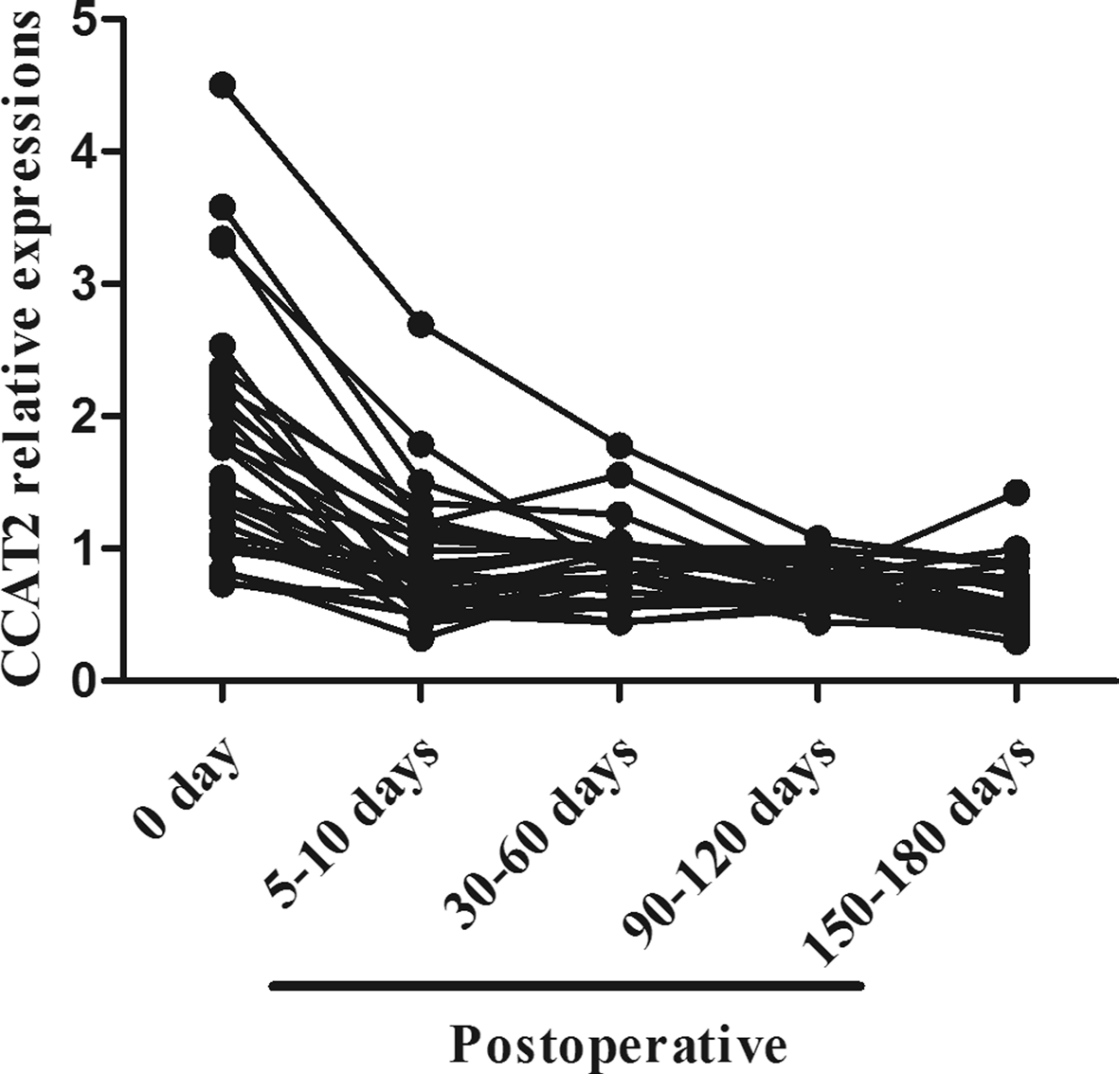
A straight line diagram of serum CCAT2 relative expression in the 30 follow-up primary CC patients

Pre- and postoperative CCAT2 was measured in 30 of the 180 primary CC who received surgery and were followed up. CCAT2 was determined before surgery (day 0) in these patients, ranging from 0.732 to 4.5 (median 1.651). At 5–10 days after surgical intervention, a significant decreasing was observed in these subjects. CCAT2 ranged from 0. 325 to 2.694 (median 0.786). At 30–60 days after surgery, CCAT2 ranged from 0.444 to 1.779 (median 0.823). CCAT2 ranged from 0.442 to 1.079 (median 0.682) during 90–120 days post-operation, and between 0.298 and 1.421 (median 0.585) during 150–180 days post-operation (Fig. 6). There was a general trend that CCAT2 was significantly higher before surgery, and decreased progressively in the follow-up period after surgery.

### Prognostic value of CCAT2 in CC patients

**Fig. 7:**
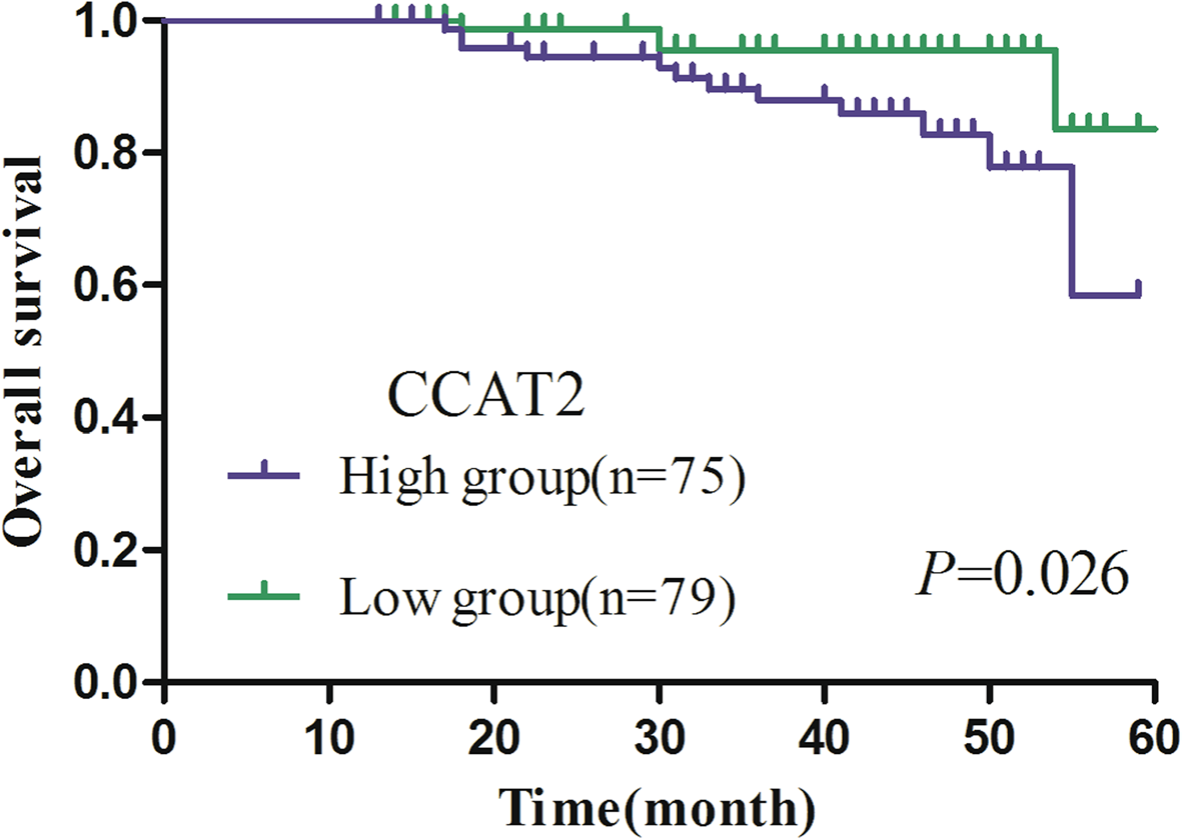
A Kaplan-Meier curve of CCAT2 for the overall survival of 154 patients with cervical cancer (CC)

We compared the overall survival times between 154 CC patients who expressed high or low expression levels of CCAT2 and SCC based on extensive clinical follow-up data. A Kaplan-Meier survival curve showed that the overall survival rate of CC patients in high CCAT2 expression group markedly decreased as compared with that of low CCAT2 expression group (*P* = 0.026) (Fig. 7). In addition, univariate analysis and multivariate analyses were per-formed by a Cox proportional hazards regression model to further assess the prognostic value of CCAT2. In the univariate analysis, FIGO stage (*P* = 0.021), lymph node metastasis (*P* = 0.001) and CCAT2 (*P* = 0.032) were associated with OS**.** In the multivariate analysis, lymph node metastasis (HR = 0.553, 95%CI: 1.684–14.690, *P* = 0.004) were independent factors associated with OS, but the influence of CCAT2 (HR = 0.654, 95%CI: 0.737–9.575, *P* = 0.135) on OS was lost (Table 4). Thus, our results may indicate that CCAT2 was not an independent prognostic factor for CC.

**Table 4:** Univariate multivariate analysis of prognostic factors on OS by the Cox regression model

Variables	Univariate analysis			Multivariate analysis		
	HR	95% CI of HR	*P* value	HR	95% CI of HR	*P* value
Age	0.142	0.450-3.251	0.706			
Tumor size	0.265	0.472-3.620	0.607			
FIGO stage	5.310	1.215-11.113	0.021*	0.671	0.773-10.727	0.115
pathological types	0.549	0.183-2.151	0.459			
Lymph node metastasis	15.260	2.672-19.329	0.001*	0.553	1.684-14.690	0.004*
CCAT2	4.606	1.114-10.774	0.032*	0.654	0.737-9.575	0.135
SCC	0.042	0.407-3.025	0.838			

*P <0.05

## Discussion

Tremendous progress has been made in the research of lncRNAs. Scientists have discovered that lncRNAs are functional transcripts that play key roles in gene regulation and therefore are regarded as new regulators of gene expression. In addition, they are closely related to tumor development and progression [8]. Kim et al. [9] found that HOXA11-AS expression in CC patients was higher than that in the control group. In vitro experiments showed that HOXA11-AS overexpression promoted cell proliferation, migration and invasion, while HOXA11-AS knockout inhibited these biological properties. Jin et al [10] found that the expression level of TCONS_00026907 in CC tissue was significantly higher than that in the para-carcinoma tissue, and that patients in the high-expression group had a lower survival rate, while silencing TCONS_00026907 expression and overexpressing miR-143-5p inhibited CC cell proliferation, migration and invasion, and promoted cell apoptosis. Hu et al. [11] reported significantly high expression of taurine-upregulated gene 1(TUG1) in the CC tissue and that was correlated with tumor size, FIGO stage and LNM. Chen et al. [12] found that high expression of lncRNA cervical carcinoma high-expressed 1 (CCHE1) in CC tissue was correlated with FIGO stage, tumor size, LNM and human papillomavirus. Kaplan-Meier survival curve showed that patients with low CCHE1 levels had better OS and recurrence-free survival (RFS).

CCAT2 is found to play an oncogene role in multiple cancers. Zhao et al. [13] reported that the nucleus and cytoplasmic β-catenin ptoein level in CCAT2 group was reduced and the Wnt signaling pathway was inhibited significantly, which had a synergistic effect with the Wnt signal inhibitor FH535. CCAT2 promoted the occurrence of non small-cell lung cancer (NSCLC) by regulating the Wnt/β-catenin signaling pathway. Wang et al. [14] reported that CCAT2 promoted epithelial-mesenchymal transition (EMT) of gastric cancer cells through downregualting the expression of E-cadherin and upregulating Zinc finger E-box binding homebox 2 (ZEB2), Vimentin and N-cadherin. However, the clinical value of CCAT2 in cancers remains unclear.

The result of RT-qPCR in the present study showed that the relative expression of serum CCAT2 was 1.616 in 180 primary CC patients, 0.795 in 80 CIN patients, and 0.727 in 100 health controls. Mann-Whitney U test showed that the serum CCAT2 relative expression level in primary CC patients was significantly higher than that in CIN patients and healthy controls (*P <* 0.001). Wang et al. [15] used tumor xenografts and immunohistochemical methods to determine the effect of CCAT2 gene knockout on tumor growth in vivo. The expression of CCAT2 was up-regulated in CC cells and tissues. The cholecystokinin (CCK8) result by Wu et al. [16] showed that CCAT2 knockout inhibited the proliferation of HeLa, CaSki and SiHa cells and promoted the proliferation and survival of CC cells. Łaźniak et al. [17] observed that the G variant of CCAT2 rs6983267 SNP induced cervical squamous cell carcinoma cells to diffuse to the surrounding tissues and promoted the rapid growth of low-grade tumor cells. All these findings provide theoretical clues to support the possibility of CCAT2 as an adjuvant diagnostic marker for CC. In addition, we found that CCAT2 relative expression was positively correlated with tumor FIGO stage, SCC-Ag and LNM (all *P* < 0.05), suggesting that CCAT2 expression level may prove to be an adjuvant marker for monitoring the degree of malignancy and disease progression.

CCAT2 relative expression was correlated with the SCC content in primary CC patients (*P* < 0.0001, *r*^2^ = 0.120) and not with the CA125 content (*P* = 0.926, *r*^2^ < 0.0001). The AUC value of CCAT2 in differential diagnosis between primary CC patients and healthy controls was 0.897 (95%CI:0.862–0.933) and the cutoff value was 1.102; under this critical value, the sensitivity, specificity, accuracy, positive prediction and negative prediction were 73.33, 87.00, 78.21, 91.03 and 64.44% respectively, indicating that CCAT2 has a relatively high sensitivity and specificity. Next a combined analysis of CCAT2, CA125 and SCC was performed using a tandem model of the three markers. The sensitivity, specificity, accuracy, positive prediction and negative prediction of this panel in differentiating the healthy controls were 94.44, 96.00, 95.00, 97.70 and 90.57%. More importantly, combination detection of CCAT2, CA125 and SCC could greatly improve the diagnostic efficiency of primary CC.

The median value of serum CCAT2 relative expression in 165 postoperative CC patients was 0.790, showing a significant decreasing as compared with that in the preoperative CC patients. The median of CCAT2 relative expression in the 44 recurrent and metastatic CC patients was 1.752, showing significant increasing from that in the primary CC patients and postoperative CC patients, suggesting that serum CCAT2 has certain significance in assessing the prognosis of CC patients.

Finally, we assessed the postoperative dynamic change of CCAT2 in 30 of the 180 primary CC patients through 6-month follow-up observation. The results showed that there was a significantly decreasing tendency in CCAT2 expression during D5–10 post-operation, which may be because the serum content of CCAT2 was decreased after resection of the tumor. Serum CCAT2 showed a slowly decreasing tendency during D30–180 post-operation. In all but one patient, imaging and clinical analysis suggested the possibility of cancer recurrence. Therefore, detection of dynamic change in serum CCAT2 could be used to dynamically monitoring the postoperative prognosis of surgical treatment in CC patients, though larger-sample studies are required to verify our conclusion. In the prognostic aspect, Kaplan-Meier survival curve showed that the overall survival rate of CC patients in high CCAT2 expression group markedly decreased as compared with that of low CCAT2 expression group. Univariable analysis showed that FIGO stage, Lymph node metastasis and CCAT2 were all significantly associated with OS.

CCAT2 was also found to be highly expressed in pancreatic ductal adenocarcinoma, ovarian cancer tissues and bladder cancer [18–20]. Above all, serum CCAT2 relative expression is to some extent associated with the development and progression of CC, suggesting that CCAT2 may prove to be an important biomarker. Analysis and assessment of the CCAT2 value in treatment and prognostic prediction of CC showed that CCAT2 detection in combination with CA125 and SCC could improve the diagnostic efficiency of CC. Our study also lays a foundation for further clarification of the action mechanism of CCAT2 in CC. It is well known that human papillomavirus (HPV) has been identified as the main factor leading to cervical cancer. Many patients with cervical cancer are positive for high-risk HPV. Qu et al. [21] found that lncRNA small nucleolar RNA host gene 8 (SNHG8) recruited enhancer of zeste homolog 2(EZH2) to downregulate reversion-inducing cysteine-rich protein with Kazal motifs (RECK) expression, leading to HPV-induced CC aggravation. This will be a good idea for the future study of the mechanism between CCAT2 and HPV in cervical cancer.

Nevertheless, the above results have some limitations. First, all samples were from the same hospital and further prospective multicenter studies will be needed. In addition, our assessment was based on the relative expression of CCAT2 in serum, and its expression in primary CC tumor tissues and cells remains to be further studies for the sake of providing more solid evidence for early diagnosis and prognostic prediction of CC.

## Data Availability

Data sharing is not applicable to this article as no datasets were generated or analysed during the current study.
